# Integrated Convolution and Attention Enhancement-You Only Look Once: A Lightweight Model for False Estrus and Estrus Detection in Sows Using Small-Target Vulva Detection

**DOI:** 10.3390/ani15040580

**Published:** 2025-02-18

**Authors:** Yongpeng Duan, Yazhi Yang, Yue Cao, Xuan Wang, Riliang Cao, Guangying Hu, Zhenyu Liu

**Affiliations:** 1 College of Information Science and Engineering, Shanxi Agricultural University, Taigu 030801, China; duan86998310@163.com (Y.D.); cy0615cy@126.com (Y.C.); 2 College of Agricultural Engineering, Shanxi Agricultural University, Taigu 030801, China; 13753412842@163.com (Y.Y.); 16635048382@139.com (X.W.); 3 College of Animal Science, Shanxi Agricultural University, Taigu 030801, China; cao13934643945@126.com (R.C.); huguangying1968@163.com (G.H.); 4 Dryland Farm Machinery Key Technology and Equipment Key Laboratory of Shanxi Province, Taigu 030801, China

**Keywords:** sow pseudo-estrus, sow estrus, YOLO, sow vulva, machine vision

## Abstract

Accurate estrus detection and optimal insemination timing are crucial for improving sow productivity and enhancing farm profitability in intensive pig farming. However, sows’ estrus typically lasts only 48.4 ± 1.0 h, and interference from false estrus further complicates detection. This study proposes an enhanced YOLOv8 model, Integrated Convolution and Attention Enhancement (ICAE), for vulvar detection to identify the estrus stages. This model innovatively divides estrus into three phases (pre-estrus, estrus, and post-estrus) and distinguishes five different estrus states, including pseudo-estrus. ICAE-YOLO integrates the Convolution and Attention Fusion Module (CAFM) and Dual Dynamic Token Mixing (DDTM) for improved feature extraction, Dilation-wise Residual (DWR) for expanding the receptive field, and Focaler-Intersection over Union (Focaler-IoU) for boosting the performance across various detection tasks. To validate the model, it was trained and tested on a dataset of 6402 sow estrus images and compared with YOLOv8n, YOLOv5n, YOLOv7tiny, YOLOv9t, YOLOv10n, YOLOv11n, and the Faster R-CNN. The results show that ICAE-YOLO achieves an mAP of 93.4%, an F1-Score of 92.0%, GFLOPs of 8.0, and a model size of 4.97 M, reaching the highest recognition accuracy among the compared models, while maintaining a good balance between model size and performance. This model enables accurate, real-time estrus monitoring in complex, all-weather farming environments, providing a foundation for automated estrus detection in intensive pig farming.

## Simple Summary

Timely sow estrus detection and insemination are crucial for farm productivity, yet challenges remain due to pseudo-estrus interference and short estrus duration. We propose ICAE-YOLO, a novel model that enhances feature extraction and bounding box regression for accurate estrus identification. Dividing estrus into the pre-, during, and post-estrus phases, this model distinguishes five estrus states, including pseudo-estrus and anestrus. ICAE-YOLO achieved the best recognition performance, while balancing model efficiency and performance, offering a new method for precise estrus detection.

## 1. Introduction

Sows play a crucial role in large-scale pig farming, where accurate and timely estrus detection is essential for farm managers. It enables the effective control of the breeding cycle, the precise scheduling of mating, the prevention of mating errors or missed opportunities, a reduction in the non-productive period, and lower breeding costs, all of which contribute to improved sow farrowing rates and enhanced reproductive efficiency. These factors directly influence the economic performance of pig farms and are the key to the success of pig farming operations [1]. Generally, a normal sow’s estrous cycle ranges from 17 to 25 days [2], with an average of 21 days [3]. However, each estrus episode typically lasts only 48.4 ± 1.0 h [1]. Based on the pregnancy rates at different estrus stages, the changes in vulvar volume and color, and the responses to external stimuli, sows undergo three estrous phases: pre-estrus, estrus, and post-estrus. In terms of specific signs, pre-estrus sows exhibit restlessness, a marked decrease in feed intake, and the swelling of the vulva (which peaks on day 1 of estrus) [4], but they resist mating and tend to move away when approached. During estrus, the sow’s vulva shows visible, sticky discharge, the ears are erect (most noticeable in gilt pigs), and the sow remains still when mounted by other sows (standing heat reaction), which also occurs when the sow’s back is pressed. At this point, the mating success rate is highest. In the post-estrus phase, the vulva begins to shrink, its color fades, feed intake normalizes, and the sow is calm, while the standing heat reaction disappears, and she refuses mating. To achieve optimal conception rates and minimize rebreeding, artificial insemination should be conducted during the optimal breeding window [5], which enhances both the litter size and the piglet survival rates [6,7].

Pseudo-estrus is a common symptom exhibited by sows in pig farming, characterized by behavioral and physical changes—particularly in vulvar shape and coloration—that closely resemble true estrus, especially during the pre-estrus and mid-estrus phases. However, unlike normal estrus, pseudo-estrus occurs without ovulation [8]. The presence of pseudo-estrus can disrupt breeding plans and is often accompanied by adverse symptoms, posing significant challenges for farmers. Multiple factors contribute to pseudo-estrus, including severe deficiencies in proteins and vitamins B1/B2, reproductive organ diseases, harsh environmental conditions, and mycotoxin poisoning. Among these, Zearalenone (ZEN) toxicity is a primary cause [9]. ZEN exhibits luteotropic properties, and the excessive intake of ZEN-contaminated feed during estrus can induce pseudo-estrus [10,11]. In newly pregnant sows, pseudo-estrus may create a false perception of gestation, leading to management difficulties. Forced breeding at this stage can result in vaginal opening, vulvar swelling, early embryonic mortality, fetal deformities, abortion, premature birth, stillbirth, and even severe reproductive tract prolapse. If pseudo-estrus is not promptly identified and sows continue to consume high-ZEN diets during lactation, it can lead to reduced milk production or complete agalactia, negatively affecting weaning weights and piglet immunity. Additionally, ZEN exposure prolongs the weaning-to-estrus interval, decreases the litter sizes [12], and increases the incidence of congenital deformities. Post-weaning, sows may experience a high return-to-estrus rate, exhibit pseudo-estrus symptoms, and suffer a 12% reduction in conception rates. Furthermore, ZEN exposure induces follicular degeneration, leading to higher culling rates and reduced sow longevity. The chronic ingestion of ZEN-contaminated feed may cause ovarian atrophy, prolonged estrous cycles, or even anestrus [13]. Piglets born to ZEN-exposed sows often exhibit feminization, reduced survival rates, and increased morbidity [14,15], along with conditions such as splay-legged syndrome and vulvar swelling. Consequently, pseudo-estrus—particularly ZEN-induced pseudo-estrus—poses severe threats to sows’ health, directly impacting the economic efficiency of swine production and presenting substantial management challenges. Timely differentiation between true and false estrus enables the implementation of appropriate breeding strategies to maximize litter sizes and production efficiency, while early intervention in addressing the underlying causes of pseudo-estrus helps prevent more severe economic losses.

In medium-tosmall-scale pig farms, estrus detection is typically performed by trained personnel through routine checks, employing methods such as visual observation, the back pressure test (BPT) [16], boar exposure testing, and body temperature monitoring. In highly automated farms, some operations utilize automated estrus detection systems, such as temperature monitoring via ear tags or implantable sensors, to reduce labor dependency. However, due to the cost constraints, technological maturity, and data processing limitations, manual estrus detection remains the primary method in most farms. For large-scale farms, manual detection is time-consuming and labor-intensive, falling short of the modern livestock production demands [17]. In terms of automated estrus detection, Ziteng Xu et al. [18] demonstrated that vulvar volume serves as a reliable indicator of estrus. Their study revealed that while sows with larger vulvar volumes exhibit a smaller percentage increase in vulvar size before and after estrus, they still undergo significant changes prior to estrus. Based on this principle, other researchers proposed an automated estrus detection method utilizing LiDAR cameras to monitor vulvar swelling pre- and post-estrus. The experimental results indicated that the LiDAR measurement error was 3.4 ± 3.0 mm, demonstrating promising detection accuracy. Ji Hwan Lee et al. [19] observed that both the standing time and the vulvar temperature increase during estrus. Leveraging this observation, they installed an ultrasonic sensor array near the pig pens and positioned digital infrared thermography devices on the sows’ tails to collect the data during estrous and non-estrous periods. Their approach, integrating ultrasonic sensors with digital infrared thermography, effectively identified estrus, providing a solid foundation for precise artificial insemination scheduling.

The non-contact, stress-free health monitoring of pigs has become a focal point of global research [20,21,22]. With declining hardware costs and advancements in deep learning, computer vision technologies are increasingly being applied to non-contact estrus detection in sows. Haibo Zheng et al. [23] developed an enhanced FD-YOLOV5s model incorporating feature fusion and dilated convolution utilizing infrared thermal imaging to automatically extract vulvar temperature variations for estrus detection. Their model achieved an impressive mean average precision (mAP) of 99.1%, an average frame rate of 156.25 fps, and a compact model size of 3.86 MB. Kaidong Lei et al. [24] proposed an intelligent mobile monitoring system for detecting estrus in weaned sows, simulating the auditory, olfactory, and tactile cues of a real boar. By employing a deep belief network (DBN), a sparse autoencoder (SAE), and a support vector machine (SVM), they successfully identified the interactions between estrous sows and the bionic boar, achieving recognition accuracies of 96.12%, 98.25%, and 90.00%, respectively. Yuan Wang et al. [25] developed a lightweight deep convolutional neural network algorithm, MobileNetV3-ResNet, to address the challenge of excessive model parameters, which often hinders real-time performance and deployment [26]. By analyzing both short- and long-frequency acoustic data, their model determined estrus status with an accuracy of 97.12%, a Recall rate of 97.34%, an F1-Score of 97.59%, a precision of 97.52%, and a model size of 5.94 MB, demonstrating the best overall performance.

According to the definition by the international organization SPIE, a small object is defined as one with an area of fewer than 80 pixels in a 256 × 256 image, which corresponds to less than 0.12% of the total image area [27]. In the field of object detection, the COCO dataset defines small objects as those smaller than 32 × 32 pixels. From the perspective of detecting the vulva of sows, in an input image of 640 × 640 pixels, the vulvar region in the dataset constructed for this study generally occupies less than approximately 27 × 27 pixels, which is about 0.18% of the total image area. Therefore, it can be considered a small-object detection challenge. Additionally, while the existing studies can determine whether a sow has entered estrus, they struggle to accurately distinguish specific estrous stages and differentiate between true and false estrus. This limitation constrains a deeper understanding and the refined management of sow reproductive behavior. To address the need for precise estrus inspection, this study proposes an improved YOLOv8 model for detecting pseudo-estrus in sows. The enhanced model not only accurately identifies sows in a pseudo-estrus state, but also timely determines the specific estrus stage of sows in normal estrus, providing strong support for precise insemination and breeding. The model incorporates three attention enhancement modules—CAFM, DDTM, and DWR—and introduces a novel bounding box regression loss function, Focaler-IoU. These attention modules, combined with the deep convolution and network architecture design, effectively improve the model’s performance in detecting the small target of a sow’s vulva. Given the functions and effects of the improved modules, this method is termed Integrated Convolution and Attention Enhancement (ICAE), and the enhanced model is named ICAE-YOLO. The primary contributions of this study are as follows:

I. This study investigates the symptoms, the vulvar changes, the hormone level variations, and the vulvar characteristics during normal estrus in sows, establishing a sow estrus dataset that includes five states: pre-estrus, estrus, post-estrus, pseudo-estrus, and anestrus.

II. To address the small-target detection challenge of sow vulva images, three attention enhancement mechanisms (CAFM, DDTM, and DWR) and a bounding box regression loss function (Focaler-IoU) are incorporated into YOLOv8. The effectiveness of these methods in improving the model’s performance is also explored.

III. ICAE-YOLO is compared with the mainstream object detection models, including YOLOv8n, YOLOv5n, YOLOv7tiny, YOLOv9t, YOLOv10n, YOLOv11n, and the Faster R-CNN, to evaluate its advantages in sow estrus detection over the existing models.

This study consists of two phases: model training and cross-scenario validation. The performance of ICAE-YOLO on the sow estrus dataset was determined during the model training phase, while the vulva image data collected in the cross-scenario validation phase (Section 3.4) were solely used to assess ICAE-YOLO’s performance across different farming environments.

## 2. Materials and Methods

### 2.1. Experimental Data

#### 2.1.1. Sow Estrus Data Collection

**Figure 1:**
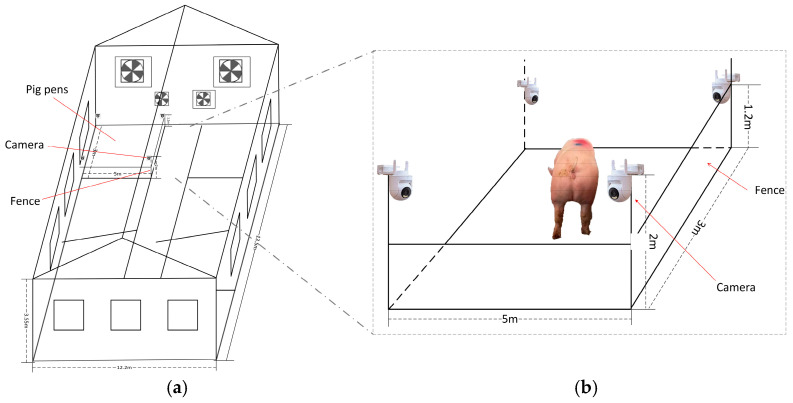
Data acquisition process. (**a**) Structure of the pig farm; (**b**) data collection process.

Data collection for the model training phase was conducted at a pig farm in Xiashan Village, Yong’an Town, Fenxi County, Shanxi Province, China (longitude: 111.603493, latitude: 36.649719). The farm was divided into eight separate areas, each measuring 3 m × 5 m, with a total area of 120 m^2^. The farm layout is illustrated in Figure 1a.

For the experiments, 26 Yorkshire pigs (also known as Large White, scientific name Yorkshire) aged 6–7 months were selected as the research subjects. Their physiological health was continuously monitored, and the data were collected throughout this study. These sows were group-housed in eight separate areas without contact with boars. The data collection period spanned from 3 June 2023 to 26 September 2023, with daily sampling occurring from 7:00 AM to 10:00 PM. This ensured that the dataset encompassed images of sows under various lighting conditions, postures, and time periods throughout the day, allowing for the comprehensive and health-assured recording of complete estrous cycles. The Large White breed was chosen due to its high feed conversion efficiency, high slaughter rate, strong adaptability, and widespread presence in China and the major pig-breeding countries worldwide. It is recognized as one of the most well-known and widely distributed lean-type pig breeds globally. The selection of 6–7-month-old sows was based on the fact that their reproductive organs and functions are fully developed, making them capable of normal reproduction (i.e., reaching sexual maturity).

To capture the vulva of the sows from different angles and postures, while ensuring image clarity, four cameras were positioned at the corners of the pig pen, approximately 80 cm above the fence height. The camera placement is shown in Figure 1b, with Xiaomi CW500 cameras (model MJSXJ07HL, Xiaomi Corporation, Beijing, China) used for the task. To increase the dataset’s diversity, some images were also captured using handheld devices from multiple angles. A total of 219 video clips from 26 pigs were collected during the data collection phase, each ranging from 5 to 60 s in length, with a frame rate of 30 fps. These videos covered all the five stages of sow estrus: false estrus, non-estrus, pro-estrus, estrus, and post-estrus. Frame extraction technology was used to sample one frame every 15 frames, resulting in a total of 3172 images.

#### 2.1.2. Data Classification

Due to hormonal fluctuations in the sows before and after estrus [28], this study employed timed hormone concentration sampling to determine estrous cycle stages and construct a control dataset for natural and false estrus. Blood samples were collected via ear vein puncture to minimize stress. Samples were taken daily at 6:00 a.m., 10:00 a.m., 2:00 p.m., and 6:00 p.m., with approximately 5–8 mL of blood collected per session and temporarily stored in vacuum tubes containing anticoagulants. After collection, the samples were rapidly refrigerated and transported to the laboratory within one hour. Hormonal concentration changes were measured using radioimmunoassay (RIA), with serum luteinizing hormone (LH), follicle-stimulating hormone (FSH), and progesterone (P4) quantified via the RIA standard curve methodology. All the samples underwent repeated measurements, revealing a distinct hormonal pattern in natural estrous cycles. The FSH levels increased before estrus, while P4 and LH remained at the baseline; during mid-estrus, LH surged sharply; post-estrus, the P4 levels gradually increased, while LH returned to the baseline. This hormone-based classification method provided an objective standard for estrous phase labeling in the dataset.

To obtain vulvar images of sows in a false estrus state, this study employed a zearalenone (ZEN)-induced protocol, which temporarily elicited estrus-like behavioral traits. Five of the twenty-six sows were continuously fed ZEN-contaminated feed to induce false estrus, and classification was also validated via blood analysis. The results indicated that in the naturally cycling sows, the FSH levels increased during pro-estrus, LH peaked during estrus, and P4 rose in the luteal phase. However, ZEN exposure resulted in no significant FSH increase, or only slight fluctuations, the absence of an LH peak, and either no significant P4 change or a slight decline. These hormone level discrepancies provided robust scientific evidence for distinguishing false estrus.

A veterinary expert team from Shanxi Agricultural University, consisting of five specialists, was involved throughout the data classification and annotation process during the model training phase. All the members specialize in animal-related research. Two experts primarily focus on hormonal regulation of the sow estrus cycle, while the other three have over ten years of experience in livestock management and behavioral studies. Since the sows in false estrus exhibited hyperactivity, the rejection of mounting pressure, and a normal appetite—differentiating them from true estrus—the experts integrated the behavioral traits, symptoms, and environmental responses to assist in classifying false estrus data. Estrous phase determination was primarily based on the blood test results, while the experts annotated the images to ensure the accurate and complete labeling of the vulvar region in the training dataset. Given the absence of significant hormonal fluctuations during false estrus, image classification was conducted using a dual-validation approach, combining behavioral observation with serum hormone analysis. Video surveillance and on-site monitoring documented standing reflexes, appetite, locomotion patterns, and other estrus-related behaviors, which were cross-referenced with the blood test results. After independent annotation, a majority voting method ensured consistency. If three or more experts agree on the classification judgment of an image, it is classified into that category; otherwise, the blood test results are used as the final criterion. To quantify annotation reliability, inter-expert agreement was assessed using Cohen’s Kappa (k) coefficient. The results showed a k-value of 0.94 for false estrus classification, indicating a high level of agreement (k > 0.90 signifies strong consistency).

#### 2.1.3. Data Set Construction

To avoid excessive similarity between adjacent frames, the Structural Similarity Index (SSIM) [29] was used to eliminate redundant images. The core calculation process of SSIM is shown in Equation (1).

(1) SSIM(X,Y)=L(X,Y)×C(X,Y)×S(X,Y)

In Equation (1), *L*(*X, Y*) is luminance comparison, *C*(*X*, *Y*) is contrast comparison, and *S*(*X*, *Y*) represents structure comparison. When it is determined that α=β=γ=1 and C3 = C2/2, SSIM can be simplified to Equation (2):

(2) $$SSIM(X,Y)=\frac{\left(2u_{X}u_{Y}+C_{1})(2\sigma_{XY}+C_{2}\right)}{\left(u_{X}^{2}+u_{Y}^{2}+C_{1})(\sigma_{X}^{2}+\sigma_{Y}^{2}+C_{2}\right)}$$

**Figure 2:**
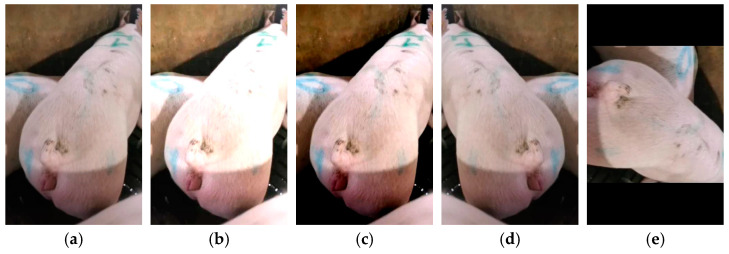
Data enhancement. (**a**) Original image; (**b**) brightness enhancement; (**c**) contrast enhancement; (**d**) random flipping; (**e**) random rotation (with cropping).

Images that were difficult to distinguish due to camera malfunctions, motion blur, extreme lighting conditions in the pig house, or occlusions by pigs, making classification and annotation impossible, were classified as extreme data and manually removed. After a series of screening, a total of 2134 images were retained. To enhance dataset diversity, data augmentation techniques were applied, including brightness adjustment, contrast enhancement, random rotation, and flipping. These augmentation operations expanded the original dataset threefold, as illustrated in Figure 2.

**Figure 3:**
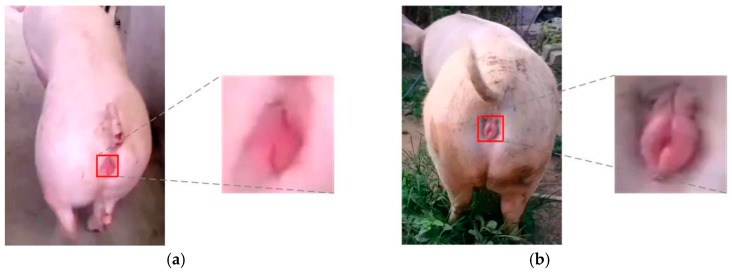
Vulva images of sows in true and pseudo-estrus. (**a**) Pre-estrus sow vulva; (**b**) vulva of sow in pseudo-estrus due to ZEN intoxication.

**Figure 4:**
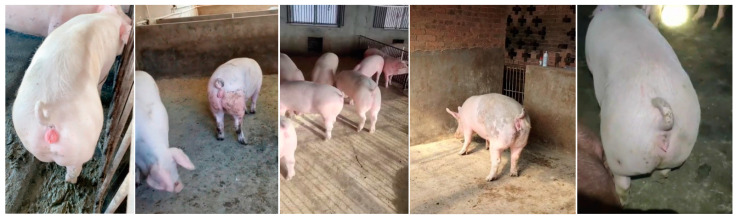
Partial images in estrus dataset of sows.

Following augmentation, a sow estrus dataset comprising 6402 images was obtained. This dataset includes vulvar images corresponding to the five estrous stages: false estrus, anestrus, pro-estrus, estrus, and post-estrus. The dataset was partitioned using random sampling, with a training-to-testing-to-validation ratio of 7:1:2. Specifically, 4481 images were allocated to the training set, 640 to the testing set, and 1281 to the validation set. The distribution of the sow estrus dataset is presented in Table 1. Representative vulvar images of the sows in natural pro-estrus and false estrus induced by zearalenone poisoning are shown in Figure 3, while sample images from the dataset are displayed in Figure 4.

### 2.2. Sow Estrus Recognition Model ICAE-YOLO

YOLOv8 [30], released in 2023 by Ultralytics—the developer behind YOLOv5—is a state-of-the-art (SOTA) model designed for various computer vision tasks, including object detection, segmentation, pose estimation, tracking, and classification. To accommodate different application requirements, five scaled versions of YOLOv8 are available based on model size: YOLOv8n (nano), YOLOv8s (small), YOLOv8m (medium), YOLOv8l (large), and YOLOv8x (extra-large) [31].

In terms of performance, YOLOv8x was evaluated on the MS COCO test-dev 2017 dataset, achieving an average precision (AP) of 53.9% and a processing speed of 280 FPS on an NVIDIA A100 with TensorRT at an image resolution of 640 pixels. In comparison, YOLOv5, using the same input size, attained an AP of 50.7% [32]. Among the five versions, YOLOv8n is the most compact, featuring a significantly reduced number of parameters and floating point operations (FLOPs), while maintaining competitive accuracy. This makes it particularly suitable for resource-constrained environments, such as mobile devices and embedded systems.

**Figure 5:**
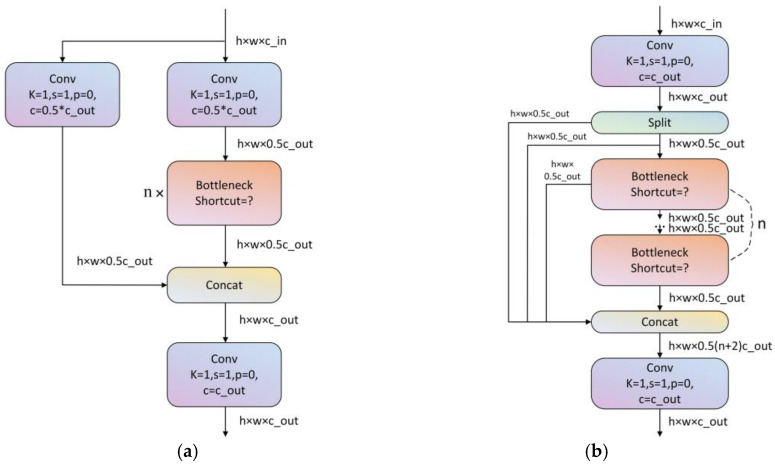
C3 and C2f modules. (**a**) Module C3; (**b**) module C2f.

The backbone and neck architecture of YOLOv8 are inspired by the ELAN design philosophy of YOLOv7. Notably, the C3 structure used in YOLOv5 was replaced with the C2f structure, which enhances gradient flow [33] (see Figure 5). Additionally, the number of channels was adjusted according to the model’s scale. Two major improvements were introduced in the head architecture compared to those of YOLOv5. First, the incorporation of a Decoupled-Head structure, which separates the classification and detection heads for improved performance; and second, the transition from an Anchor-Based to an Anchor-Free approach, which simplifies the model and enhances detection accuracy [34].

Intersection over Union (IoU) is a widely used metric for evaluating the overlap between two bounding boxes. In computer vision, IoU is primarily used for accuracy assessment and is calculated as the ratio of the intersection area to the union area between the “target box” and the “predicted box”, providing a measure of their overlap. Complete IoU (CIoU) [35] extends the standard IoU by incorporating additional factors, including the distance between the center points of the bounding boxes, as well as the differences in width and height. These enhancements enable CIoU to provide a more precise evaluation of bounding box quality and positioning.

YOLOv8 leverages CIoU and the Distribution Focal Loss (DFL) function for bounding box regression, while Binary Cross-Entropy (BCE) is used as the classification loss function to compute confidence scores. The BCE loss function is defined in Equation (3), IoU is calculated using Equation (4), DFL is computed according to Equation (5), and CIoU is determined as shown in Equation (6).

(3) $$BCEL\mathrm{oss}=-\frac{1}{n}\sum_{i=1}^{n}\left[y_{i}\times lo\mathrm{gp}(y_{i})+(1-y_{i})\cdot log(1-p(y_{i}))\right]$$

where *y_i_* is label 1 or 0, i.e., yes or no for binary classification; *p*(*y_i_*) can be interpreted as the predicted label value corresponding to the true label *y_i_*.
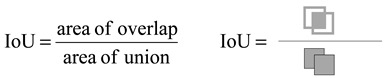
(4)

(5) $$DFL(S_{i},S_{i+1})=-((y_{i+1}-y)\log(S_{i})+(y-y_{i})\log(S_{i+1}))$$

(6) $$CI\mathrm{oU}=1-I\mathrm{oU}+\frac{\rho^{2}(b,b^{gt})}{c^{2}}+\alpha v$$

In Equation (6), IoU is the intersection and concurrency ratio, *b* and *b^gt^* denote the centroids of the two rectangular boxes, *ρ* denotes the Euclidean distance between the two rectangular boxes, *c* denotes the diagonal distance between the two rectangular boxes in the closed region, *v* is used to measure the concordance of the relative proportions of the two rectangular boxes, and *α* is the weighting factor.

#### 2.2.1. DDTM Attention Mechanism Module

**Figure 6:**
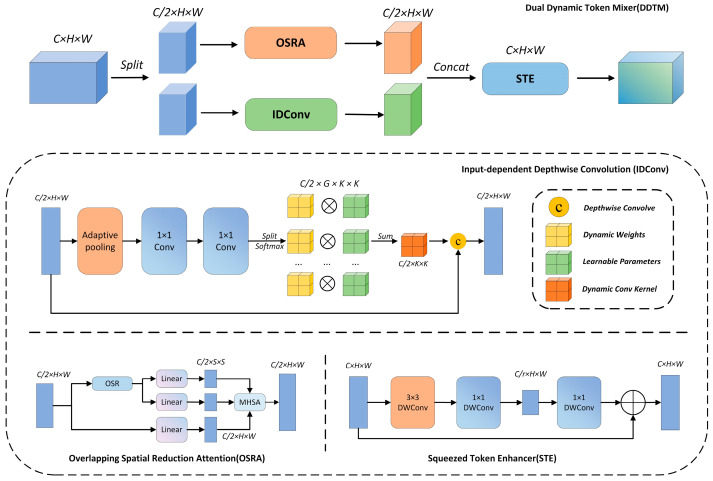
Structure of DDTM Attention module.

The recent studies have demonstrated that integrating global contextual information with local details significantly enhances model robustness and performance in complex object detection tasks. For instance, the Vision Transformer (ViT) has achieved remarkable results using a self-attention mechanism; however, it lacks effectiveness in local feature extraction. To address this limitation and better capture both global and local features, we introduce a lightweight dual dynamic token mixing network module, termed the Dual Dynamic Token Mixer (DDTM) [36]. The DDTM processes input features through two parallel streams, one employing a global self-attention module, and the other utilizing an input-dependent depthwise convolution module. The outputs are then fused to enhance contextual understanding and local feature extraction. In this study, we integrate the DDTM into two C2f modules within the YOLOv8 backbone, forming the C2fDDTMAttention module, which strengthens feature extraction and improves detection performance. The network structure of the DDTM is illustrated in Figure 6.

#### 2.2.2. CAFM Attention Mechanism Module

**Figure 7:**
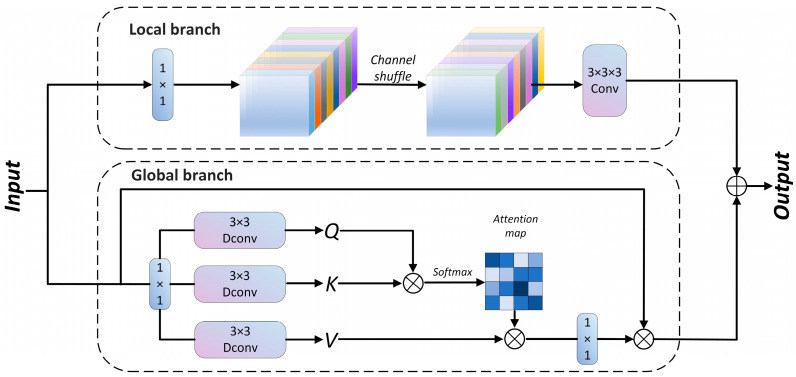
Structure of CAFM Attention module.

In object detection, the fusion of global contextual information with local features is crucial for enhancing the small-object detection performance. To further improve the modeling of global and local features, we introduce a Convolution and Attention Fusion Module (CAFM) [37], which combines convolution for local feature extraction with self-attention for global information modeling. The CAFM consists of separate branches for global and local feature processing; the convolutional branch focuses on detailed local feature representation, while the attention mechanism captures long-range dependencies. By embedding the CAFM into the YOLOv8 backbone, we enhance the network’s ability to model complex spatial relationships, thereby improving detection accuracy. The structure of the CAFM module is shown in Figure 7.

#### 2.2.3. DWR Attention Mechanism Module

**Figure 8:**
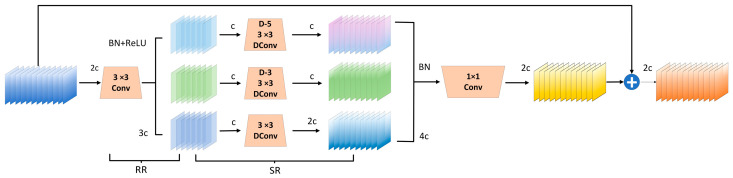
Structure of DWR module.

Dilated convolution, a technique for expanding the receptive field, has been widely applied in object detection. Given the challenge of detecting small vulvar regions in sow estrus images, we introduce a feature extraction module based on dilation-wise residual learning (Dilation-wise Residual, DWR) [38] to capture multi-scale contextual information from a single input feature map, while reducing feature extraction complexity. DWR leverages dilated convolutions to expand the receptive field, while maintaining computational efficiency. The module decomposes feature learning into regional residualization and semantic residualization; the regional residualization phase applies grouped depthwise separable dilated convolutions with varying dilation rates to improve learning flexibility, whereas the semantic residualization phase employs morphology-based filtering to extract the key semantic structures, ensuring more effective small-object detection. By integrating DWR into the YOLOv8 neck, we achieve efficient multi-scale feature fusion and enhance the model’s ability to focus on small vulvar regions during estrus detection. The DWR module structure is illustrated in Figure 8.

#### 2.2.4. Focaler-IoU

The traditional object detection loss functions often suffer from class imbalance and an uneven distribution of easy- and hard-to-process samples. For detection tasks dominated by easy-to-process samples, focusing on these samples during bounding box regression can improve the overall detection performance. However, for tasks with a higher proportion of hard-to-process samples, prioritizing hard-to-process samples in bounding box regression is essential. The existing studies have attempted to enhance regression performance by leveraging the geometric relationships between bounding boxes, but have largely overlooked the impact of sample difficulty distribution. However, variations in sample difficulty significantly influence regression accuracy. To better handle different regression samples and detection tasks, we employ a linear interval mapping method to reconstruct the IoU loss function, introducing Focaler-IoU [39]. By emphasizing regression samples of varying difficulty levels, Focaler-IoU enhances the detector’s performance across diverse detection scenarios. The mathematical formulation of Focaler-IoU is presented in Equation (7).

(7) $${\mathrm{IoU}}^{\mathrm{focaler}}=\left\{\begin{array}{ll} 0, & \mathrm{IoU}<d\\ \frac{\mathrm{IoU}-d}{u-d}, & d<<\mathrm{IoU}<<u\\ 1, & \mathrm{IoU}>u \end{array}\right.$$

where IoUfocaler is reconstructed Focaler-IoU, IoU is for the original IoU value, and [*d*, *u*] ∈ [0, 1]. By adjusting the values of *d* and *u*, IoUfocaler can be made to focus on different regression samples. Its loss is defined in Equation (8).

(8) $$L_{\mathrm{Focaler}\text{-}\mathrm{IoU}}=1-{\mathrm{IoU}}^{\mathrm{focaler}}$$

**Figure 9:**
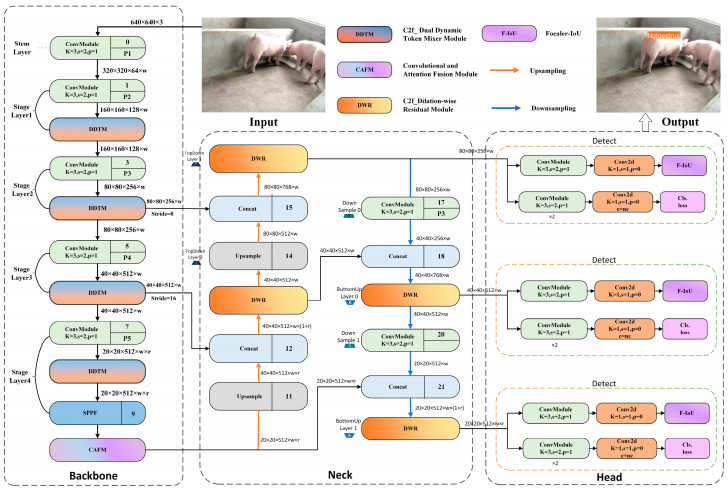
ICAEM-YOLOv8 network structure diagram.

The improved YOLOv8 network structure is illustrated in Figure 9.

### 2.3. Experimental Platform

In this study, training and prediction were performed on a laptop with the Windows system equipped with Intel Core i5-12450H @2.00 GHz and an NVIDIA GeForce 3060 laptop. The framework used is PyTorch, the integrated development environment is PyCharm, and the programming language is Python 3.8, and the experimental platform (hardware and software configuration) used in the cross-scenario validation phase in Section 3.4 was identical to that employed during the model training phase. All subsequent comparative algorithms were trained and evaluated in the same environment to ensure consistency in the experimental results. The detailed core hardware configuration of the experimental platform is shown in Table 2, and the main software information is shown in Table 3.

### 2.4. Performance Indicators

The Confusion Matrix [40] is a table used in machine learning and deep learning to evaluate the performance of classification algorithms, typically by visualizing and analyzing the results of classification models. Average Precision (AP) is a metric used to measure the performance of tasks such as object detection and image classification. In object detection, AP assesses the degree of overlap between predicted and ground truth bounding boxes, with a range from 0 to 1. A value of 1 indicates a perfect performance, while 0 indicates a poor performance. A higher AP value reflects better accuracy and Recall for the detection algorithm in the corresponding class. The evaluation metrics used in this study include AP_Pseudoestrus_, AP_Notoestrus_, AP_Preoestrus_, AP_Midoestrus_, AP_Lateoestrus_, and AP_Allclass_, which represent the average precision for the pseudo-estrus, non-estrus, pre-estrus, mid-estrus, and late-estrus phases, as well as the mean average precision (mAP) across all the five classes. Additionally, other important evaluation metrics are also discussed below.

1. Accuracy

Accuracy is the proportion of all correct predictions (including both positive and negative classes) out of the total predictions. The calculation formula is shown in Equation (9):

(9) $$A\mathrm{ccuracy}=\frac{TP+TN}{TP+FN+TN+FP}=\frac{TP+TN}{P+N}$$

2. Precision

Precision rate is the proportion of correctly predicted positive categories to the proportion of all predicted positive categories, and its calculation formula is shown in Equation (10):

(10) $$P\mathrm{recision}=\frac{TP}{TP+FP}$$

3. Recall

The Recall rate, also known as the check rate, represents the proportion of values correctly predicted for the positive category out of the total positive category. The formula is shown in Equation (11):

(11) $$R\mathrm{ecall}=\frac{TP}{TP+FN}=\frac{TP}{P}$$

4. F1-Score

Precision and Recall vary in different models. (4) The F1-Score integrates the precision and Recall of the model and is the reconciled average of these two. The F1-Score calculation process is shown in Equation (12).

(12) $$F1S\mathrm{core}=\frac{1}{\frac{1}{2}(\frac{1}{\mathrm{precision}}+\frac{1}{R\mathrm{ecall}})}$$

5. GFLOPs

In deep learning, especially when using neural networks for inference or training, computational complexity is often measured by the number of floating point operations (FLOPs). FLOPs quantify the number of floating point calculations a model requires, serving as an indirect metric for model complexity and speed. GigaFLOPs (GFLOPs) represent billions of floating point operations. As a crucial indicator for evaluating model efficiency, fewer GFLOPs generally imply faster inference speeds, reduced hardware demands, and less energy consumption. 

## 3. Results

In this study, the input image size was standardized to 640 × 640 pixels. To accelerate convergence, the initial learning rate was set to 0.01, with a weight decay coefficient of 0.0005 and a momentum factor of 0.937. The model was trained for 250 epochs with a batch size of one.

### 3.1. Ablation Experiment

**Figure 10:**
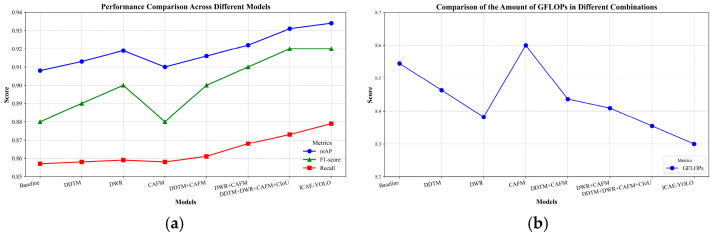
Performance improvement effect of each module combination on the algorithm. (**a**) Comparison of recognition performances; (**b**) comparison of GFLOPs.

To investigate the impact of different improvement modules on the model performance, ablation experiments were conducted by integrating the proposed four improvements with YOLOv8 in various configurations. These model combinations were trained, validated, and evaluated on the estrus dataset, with the final ablation results presented in Table 4. The performance improvements achieved by the different combinations are illustrated in Figure 10.

The ablation results demonstrate that ICAE-YOLO, which incorporates the DDTM, DWR, and CAFM attention mechanisms along with Focaler-IoU, achieves the best overall performance on the estrus dataset, with an mAP of 0.934, an F1-Score of 0.92, a Recall of 0.879, and a GFLOP of 8.0, effectively balancing the computational cost and detection accuracy. Additionally, the results highlight DWR as an outstanding enhancement module, significantly improving the model performance, as evidenced by its superior mAP of 0.919 and GFLOP of 8.3, outperforming the YOLOv8n + DDTM + CAFM combination. However, it is noteworthy that CAFM alone does not yield satisfactory improvements; while it increases the mAP by only 0.02, it also leads to a 0.2 GFLOP increase. Nevertheless, when integrated with the other modules, it contributes significantly to the overall enhancement of the algorithm, suggesting potential for further optimization in future research.

### 3.2. Results of the ICAE-YOLO Model

**Figure 11:**
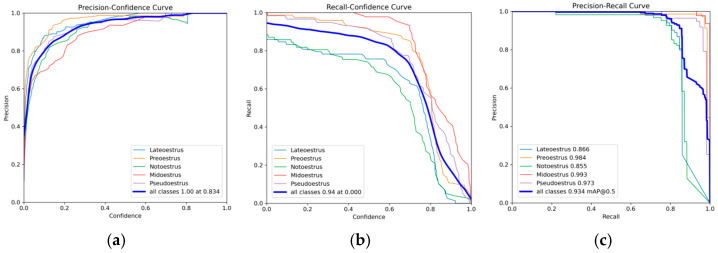
Precision curve, Recall curve, and PR curve of ICAE-YOLO. (**a**) Precision–confidence curve; (**b**) Recall–confidence curve; (**c**) precision–Recall curve.

**Figure 12:**
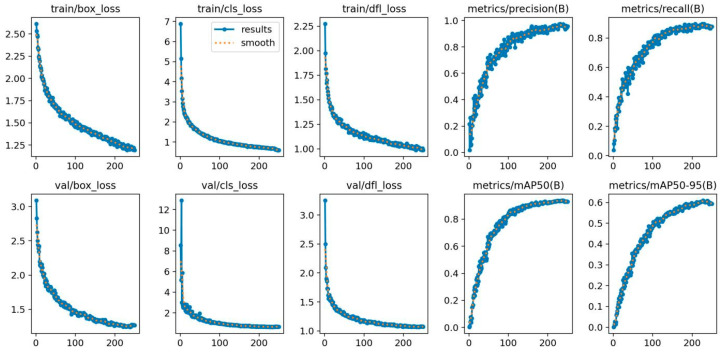
ICAE-YOLO results.

**Figure 13:**
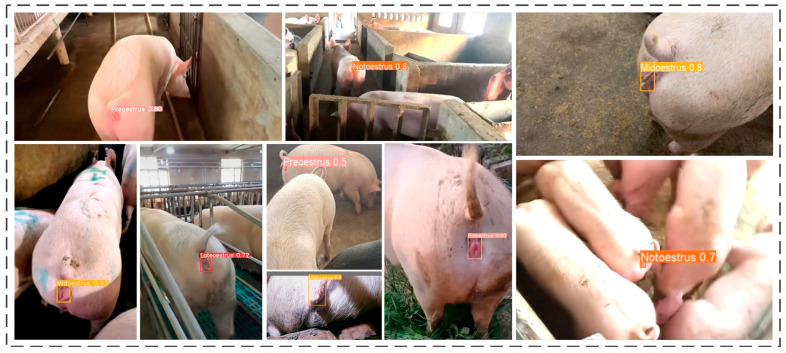
Effectiveness of ICAE-YOLO in recognizing sows in different estrous states.

The experimental results presented in Table 4 validate the effectiveness of ICAE-YOLO in accurately identifying the pseudo-estrus and various estrus phases in sows. Figure 11 illustrates the precision–confidence, Recall–confidence, and P-R curves of ICAE-YOLO, while Figure 12 provides additional detailed results. Furthermore, Figure 13 offers a focused visualization of ICAE-YOLO’s performance in recognizing the different pseudo-estrus, non-estrus, and estrus phases. As shown in Figure 12, the train/box_loss metric exhibits a rapid decline from approximately 2.5 to 1.5 within the first 100 epochs, indicating the model’s quick adaptation in accurately localizing the target bounding boxes. As training progresses, train/box_loss continues to decrease, approaching zero, which signifies the model’s high precision in predicting the sow’s vulva position within the training images. Train/cls_loss starts at around 6.9 and drops sharply to approximately 1.0 within the first 100 epochs, demonstrating the model’s rapid improvement in correctly classifying the targets within the bounding boxes. It continues to decline, converging around 210 epochs, reflecting a consistent enhancement in classification accuracy on the training set. Similarly, train/dfl_loss, initially occurring at about 2.25, decreases to approximately 1.1 within the first 100 epochs, contributing to the model’s improved confidence in bounding box coordinate predictions. It further falls below one after 240 epochs, indicating increased precision in bounding box localization on the training set.

The metrics/precision(B) and metrics/Recall(B) exhibit a continuous upward trend, improving steadily with the number of training epochs. By approximately 250 epochs, precision approaches one, highlighting the model’s enhanced ability to identify the true positives, while reducing the false positives. The Recall metric reflects the model’s increasing capacity to identify the actual positives in the estrus dataset.

The loss metrics on the validation set are critical for assessing the model performance. The val/box_loss metric decreases from approximately 3.1 at the beginning to below 1 by 250 epochs, indicating the model’s strong capability in localizing the small vulva targets in the validation set, thereby demonstrating good generalization. The convergence of val/cls_loss and val/dfl_loss further signifies the model’s improved accuracy in classifying the sows’ vulvas in the validation set and its enhanced precision in bounding box prediction.

The average precision metric, metrics/mAP50(B), at an IoU threshold of 0.5, shows a significant upward trend, steadily increasing from 0 and surpassing 0.9 after 200 epochs. This indicates an excellent performance in correctly detecting and classifying the moderately overlapping targets. Additionally, the average precision metric, metrics/mAP50-95(B), which spans IoU thresholds from 0.5 to 0.95, also demonstrates continuous improvement, exceeding 0.6 after 200 epochs. This suggests a robust performance in detecting and classifying the targets with varying degrees of overlap.

Overall, these results underscore the model’s strong recognition capabilities and generalization performance.

From Figure 13, it is evident that ICAE-YOLO accurately identifies the sows in a pseudo-estrus state by analyzing the changes in the vulvas. Additionally, for the sows in the normal estrus period, the model can further determine the specific estrus stage, providing valuable insights for breeders to take appropriate reproductive actions. This effectively accomplishes the recognition tasks proposed in this study.

### 3.3. Comparison of Target Detection Models of the Same Type

**Figure 14:**
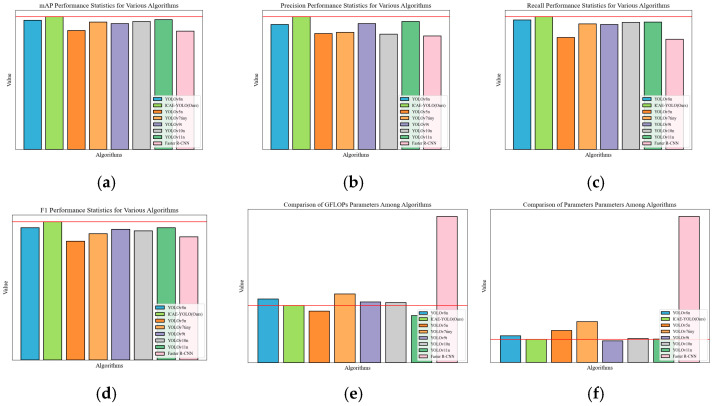
Comparison of performances of different models of algorithm. (**a**) mAP; (**b**) precision; (**c**) Recall; (**d**) F1-Score; (**e**) GFLOPs; (**f**) parameters.

To further evaluate the advantages of ICAE-YOLO in sow estrus detection compared to those of the existing object detection models, we conducted performance comparison against seven different algorithms: YOLOv8n, YOLOv5n, YOLOv7tiny, YOLOv9t, YOLOv10n, YOLOv11n, and the Faster R-CNN. The experimental comparison results are presented in Table 5, while Figure 14 provides a more intuitive visualization of the performance differences among these algorithms. The performance improvement rates of ICAE-YOLO alongside those of the other algorithms are detailed in Table 6.

**Figure 15:**
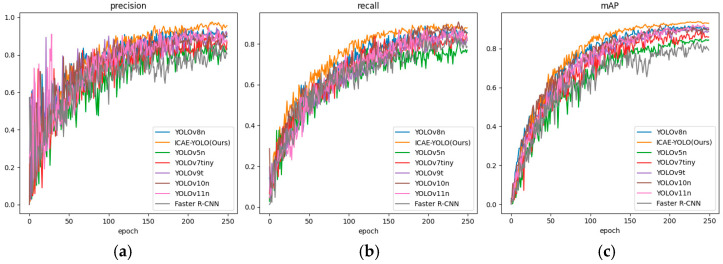
Precision, Recall and mAP curves for eight algorithmic iterative processes. (**a**) Precision; (**b**) Recall; (**c**) mAP.

The experimental results indicate that ICAE-YOLO achieves an mAP of 93.4%, with F1-Score, precision (P), and Recall (R) values of 92%, 96%, and 87.9%, respectively. Compared to those of the smallest model in the YOLOv8 series, YOLOv8n, ICAE-YOLO improves the mAP by 2.86% while reducing the GFLOPs by 10.11%. Additionally, ICAE-YOLO outperforms YOLOv5n, YOLOv7tiny, YOLOv9t, YOLOv10n, and the Faster RCNN in mAP by 11.58%, 4.24%, 5.41%, 3.89%, and 12.26%, respectively. Although ICAE-YOLO exhibits a 21.21% increase in GFLOPs compared to that of the latest YOLO11 model, it offers superior detection accuracy and parameter efficiency. Overall, ICAE-YOLO maintains the best recognition accuracy, while achieving a competitive level of model lightweighting compared to those of the seven alternative algorithms. The precision, Recall, and mAP curves during the iterative processes of all the eight algorithms are illustrated in Figure 15, while the average precision for each category and the mean average precision (mAP) are presented in Table 7.

**Figure 16:**
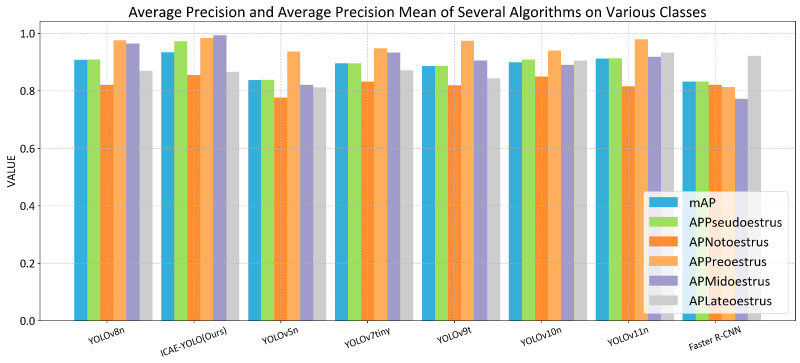
Average recognition accuracy of eight algorithms in each category.

**Figure 17:**
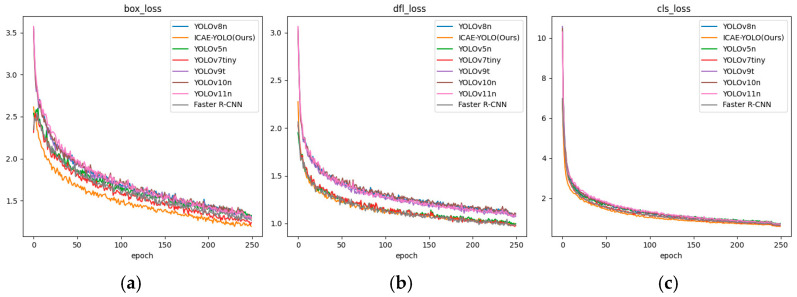
Variation in loss curves during iterations of several algorithms. (**a**) Box loss; (**b**) dfl loss; (**c**) cls loss.

As shown in Table 7, ICAE-YOLO achieved the highest average precision in detecting the pseudo-estrus, pre-estrus, and mid-estrus phases. Specifically, ICAE-YOLO attained an average precision of 97.3% for pseudo-estrus detection, surpassing YOLOv8n, YOLOv5n, YOLOv7tiny, YOLOv9t, YOLOv10n, YOLOv11n, and the Faster R-CNN by 7.04%, 14.74%, 8.59%, 9.69%, 7.04%, 6.57%, and 16.95%, respectively. For pre-estrus detection, it achieved 98.4%, with improvements of 0.82%, 3.90%, 3.79%, 1.02%, 4.68%, 0.51%, and 21.03% for the same models. In mid-estrus detection, ICAE-YOLO reached 99.3%, outperforming the seven algorithms by 3.00%, 19.49%, 6.43%, 9.60%, 11.44%, 8.05%, and 28.62%, respectively. For non-estrus detection, the model attained an average precision of 85.5%, exceeding the others by 4.14%, 8.64%, 2.76%, 4.39%, 0.58%, 4.77%, and 4.14%. However, in late estrus detection, the improved algorithm exhibited a slight decline, with reductions of 0.46%, 0.57%, 4.30%, 7.18%, and 6.07% compared to those of YOLOv8n, YOLOv7tiny, YOLOv10n, YOLOv11n, and the Faster R-CNN, respectively. This suggests potential areas for improvement in capturing subtle vulvar feature changes during late estrus. Figure 16 presents the comparative analysis of average precision and mAP across all the categories, while Figure 17 illustrates the loss curves during the iterative training process for the different algorithms.

**Figure 18:**
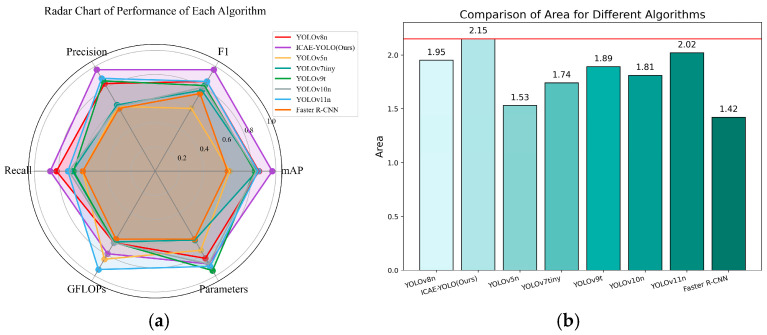
Performance radargrams of the eight algorithms. (**a**) Comparison of radar map performances; (**b**) area occupied by each algorithm in radargram.

To provide a comprehensive performance comparison, a radar chart (Figure 18) was plotted. For consistency, the GFLOPs and the parameter counts were normalized inversely, meaning that lower values indicate a higher computational load and larger parameter counts, while values closer to one represent lower computational demands and greater efficiency. The radar chart reveals that although ICAE-YOLO slightly lags behind YOLOv9t and YOLOv11n in computational efficiency, it covers a larger overall area with a well-balanced distribution across the various performance metrics, demonstrating superior overall effectiveness.

### 3.4. Real-World Scenario Evaluation

#### 3.4.1. Testing Methodology and Data Acquisition

**Figure 19:**
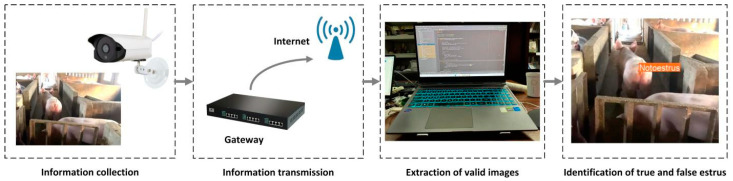
Sow estrus data collection and identification system workflow.

To validate the applicability of ICAE-YOLO in real-world scenarios, model verification was conducted in Shanxi Province, China, to evaluate its performance across the different farming environments. As shown in Table 8, the test data were collected from Duancun Village, Sengnian Town, Fenxi County, and Pu County, Shanxi Province. These data were solely used to assess ICAE-YOLO’s effectiveness in diverse real-world conditions. The validation dataset included 13 primiparous sows of three different breeds from two test sites, among which 3 sows were ZEN-induced pseudo-estrus cases, while the remaining 10 were at varying estrus stages. The dataset captured variations in posture, angles, and lighting conditions throughout the day, ensuring maximum complexity and diversity. A sow estrus data acquisition and recognition system was designed, as illustrated in Figure 19. High-definition cameras were installed on-site and connected via an intelligent gateway. The video stream captured by the cameras was wirelessly transmitted to the monitoring room for image extraction and subsequent identification by ICAE-YOLO. The camera installation positions, models, and image sampling methods were consistent with those described in Section 2.1.1, and similar images were filtered using SSIM. To ensure the reliability and accuracy of validation results, blood samples were collected from the target sows during the validation phase. Radioimmunoassay (RIA) was employed to measure the concentrations of luteinizing hormone (LH), follicle-stimulating hormone (FSH), and progesterone (P4), which served as reference indicators for estrus determination.

#### 3.4.2. Expert Collaboration and Model Validation

Model validation was conducted in collaboration with veterinary experts from Shanxi Agricultural University. Five experts independently performed estrus identification on the collected validation data alongside our proposed method. Validation faced challenges from the variations in pig breeds, the environmental conditions at the different times, and the changes in posture and angle. The model’s predictions were statistically compared with expert assessments. The experts independently evaluated all the validation images based on field observations and captured images to determine the estrus stages. Each expert classified each image into one of the predefined categories. To ensure a reliable consensus, a majority voting strategy was adopted. If at least three out of five experts assigned the same classification to an image, it was considered to belong to that category. In cases where votes were distributed across multiple categories without a clear majority, a secondary discussion was conducted to reach a final decision. The actual classification results were statistically validated against the serum test findings. The final quantitative results for both the model and expert assessments were computed using the methods outlined in Section 2.4 (Performance Indicators). The results are presented in Table 9.

From the results in Table 9, it can be seen that the model achieved satisfactory prediction results, with its precision, Recall, and F1-Scores closely matching the expert assessments, with an average error rate of −3.3786%. These evaluations demonstrate the applicability of ICAE-YOLO in real-world complex farming scenarios, while also highlighting its shortcomings. This will aid in the further improvement and optimization of the model. We believe that with continuous iteration and refinement, ICAE-YOLO will deliver an even better performance in the task of identifying true and false estrus.

## 4. Discussion

### 4.1. Discussion of Current Automated Sow Estrus Detection Methods

The performance of a sow estrus recognition model primarily depends on the quality and diversity of the dataset. However, deep learning-based automated estrus detection in sows still faces significant challenges, particularly in data acquisition. Estrus determination heavily relies on the expertise of inspectors, with hormone testing required for precise phase identification. Additionally, the short duration of estrus and occasional false estrus further complicate dataset collection and annotation, making it difficult to obtain a well-labeled dataset. Currently, no publicly available dataset exists for this task. This study summarizes the recently developed deep learning-based methods for livestock estrus detection, as detailed in Table 10.

According to the data given in Table 10, the recently developed deep learning-based estrus detection methods for livestock have mainly focused on pigs and cattle, achieving significant progress. The recognition models for pigs and cattle listed in Table 10 show mAP values higher than that of ICAE-YOLO (93.4%). Notably, FD-YOLOV5s (3.86 M) proposed by Haibo Zheng et al., E-YOLO (3.04 M) created by Zheng Wang et al. and the improved YOLOv5n (0.72 M) used by Wang Zheng et al. all have fewer parameters than ICAE-YOLO (4.97 M), demonstrating superior performances. However, these studies mainly focus on the binary classification of estrus and non-estrus. For pig breeding, inseminating during the peak estrus phase is more favorable for pregnancy. Additionally, compared to the invasive sensor-based estrus detection methods, ICAE-YOLO does not offer superior accuracy or reliability. For example, Johnson et al. [45] inserted a data logger into the vagina during the pre-estrus phase to monitor activity level changes, accurately determining estrus onset. However, ICAE-YOLO’s non-invasive method avoids intrusion and reduces the sows’ stress level.

In summary, this study precisely divides sow estrus into three phases—early, mid, and late—and identifies five distinct states, including false and non-estrus. This approach provides a more diverse and scientifically robust method for non-contact, automated sow estrus detection and inspection.

### 4.2. Analysis of Model Limitations

**Figure 20:**
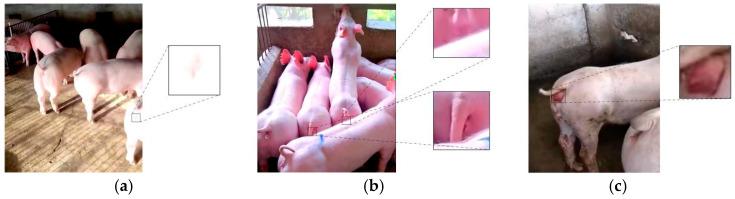
Conditions leading to model restriction. (**a**) Extreme light exposure; (**b**) shading; (**c**) bacterial vaginitis.

Although ICAE-YOLO can effectively identify sow estrus, its application as an accurate method for estrus detection in commercial farms remains a significant challenge. This challenge arises in several areas. First, external genital images of the sows in false estrus induced by ZEN were taken, and the core cause of false estrus in this study was identified as fumonisin contamination. The impact of false estrus caused by other factors, such as nutritional deficiencies (e.g., proteins or vitamins B1 and B2) and harsh environmental conditions, on the model’s performance still requires further verification. Secondly, factors such as objects or other pigs obstructing the view, tail hair self-obstruction, backlighting, and severe fecal stains can lead to decreased accuracy in the model’s localization and recognition of genital targets, sometimes resulting in model failure. (As illustrated in Figure 20a, strong lighting causes the external genitalia to blend with the background, making the shape indistinct and difficult to distinguish from the background. In Figure 20b, self-occlusion by the sow’s tail significantly reduces the vulva region’s pixel area, accounting for less than 0.05% of the entire image.) Lastly, abnormal conditions, such as vaginal inflammation, vulvar inflammation, uterine infections, and external genital injuries, causing symptoms, such as swelling, increased secretions, redness, and changes in the shape and color of the external genitalia, resemble changes during false estrus or estrus early and mid-stages (Figure 20c depicts vulvar swelling due to bacterial vaginitis), posing challenges and interference for the model in identification.

The dataset used for model training was represented by Large White pigs. It is important to note that estrus cycles, estrus duration, and genital features vary by age, breed, and exposure to boars. These factors could theoretically influence the model’s performance, and further experiments and practical analysis are needed to validate this. Additionally, in practical applications, artificial insemination remains a necessary step. Since ICAE-YOLO relies solely on images, it may detect the estrus stages slightly earlier or later than human inspection based on behavioral or multimodal data. Therefore, integrating the technical detection results with manual procedures is essential to ensure the best pregnancy rates. Further improvements can be made by adjusting algorithm detection thresholds and increasing the dataset size for training to enhance the algorithm’s reliability.

Furthermore, although ICAE-YOLO demonstrates high accuracy in estrus and false estrus detection, there are challenges to its commercialization in the pig farming industry. The primary obstacle is the initial investment cost, as implementing deep learning-based detection systems requires specialized hardware, such as high-resolution cameras, servers, and network infrastructure, which may not be economically feasible for small- and medium-sized farms. Additionally, equipment maintenance and system calibration are critical to ensuring long-term reliability, as environmental factors like dust and light changes can affect the detection performance. Another key factor is the acceptability of the technology and its integration with existing farm management practices. The traditional estrus detection methods such as back pressure tests remain widely used due to their simplicity, reliability, and cost-effectiveness, leading to resistance against adopting assisted insemination. Farms with trained personnel may perceive there to be limited added value, whereas large-scale automated farms could benefit more from ICAE-YOLO’s efficiency in reducing labor-intensive monitoring. Addressing these challenges through cost-effective implementation strategies, user-friendly system designs, and integration with the existing breeding management protocols will be crucial for promoting the widespread adoption of ICAE-YOLO in modern pig farming.

### 4.3. Discussion of the Effect of Light on the Performance of ICAE-YOLO

In practical scenario testing, sow estrus images were collected from various pig farms, breeds, and lighting conditions to validate the model’s performance. To further analyze the impact of different lighting conditions on ICAE-YOLO, the images were categorized into three groups: normal lighting, bright lighting, and low lighting. To ensure consistency and minimize the influence of sample size on the results, each category contained approximately 90 test images. The results are summarized in Table 11.

As shown in Table 11, ICAE-YOLO achieved an mAP of 96.8% under the normal lighting conditions. However, under intense lighting, the mAP dropped significantly to 84.4%, a decline of more than 12.80%. This suggests that the extreme lighting conditions have a substantial negative impact on ICAE-YOLO’s performance, even more so than the low-light conditions. In practice, extreme lighting conditions should be minimized to enhance the model’s detection performance for small vulva targets. Furthermore, this finding highlights areas where the model can be further optimized, providing valuable insights for future improvements.

### 4.4. Discussion on the Impact of Confidence Threshold on the Performance of ICAE-YOLO

In object detection algorithms, balancing the confidence threshold with detection efficiency is critical, as the threshold selection directly affects the model performance. The detailed analysis shown in Figure 11a,b reveals that as the confidence threshold increases, precision steadily improves, reflecting more reliable predictions and a reduction in false positives. The highest precision of one is achieved at a confidence threshold of 0.834. Conversely, increasing the confidence threshold leads to a steady decline in Recall, indicating that the model becomes more conservative and fails to detect some positive samples. Once the threshold surpasses 0.6, Recall declines sharply, suggesting a significant increase in missed detection. These findings demonstrate that the confidence threshold is the key parameter in practical sow estrus detection tasks. A lower confidence threshold can be selected to minimize missed detection, while a higher threshold can be used to reduce the number of false alarms. In real-world applications, the confidence threshold should be adjusted based on specific detection requirements to achieve an optimal performance.

## 5. Conclusions

This study presents an efficient automated sow estrus detection model, ICAE-YOLO, based on improvements to YOLOv8. Compared to the existing models, ICAE-YOLO is specifically designed to address the challenges posed by small sow vulva targets and complex, interfering farm environments. The improved model accurately identifies the sows in false estrus based on vulva color and appearance changes. For the sows in normal estrus, the model can also determine the three specific estrus phases, providing valuable reference information for optimal insemination timing. The enhanced model achieves a mAP of 93.4%, an F1-Score of 92.0%, a Recall of 87.9%, and the GFLOPs reduced from 8.9 in YOLOv8n to 8.0. When compared with YOLOv8n, YOLOv5n, YOLOv7tiny, YOLOv9t, YOLOv10n, YOLOv11n, and the Faster R-CNN, ICAE-YOLO demonstrates superior recognition accuracy, while maintaining a good balance between computational complexity and performance. The improved algorithm meets the demands of the real-time, all-weather monitoring of sow estrus and false estrus, representing a significant advancement in the field. This research can be applied to large-scale farms to improve animal welfare. Future work will explore the variations in vulva characteristics of sows in false estrus caused by different factors; assess the impact of breed, parity, and exposure to boars on model performance; and further validate the model’s reliability in more pilot farms, offering a more reliable scientific approach for sow estrus detection.

## Figures and Tables

**Table 1:** Distribution of sow estrus datasets.

Category	Raw Data Distribution	Total Number of Raw Data	Expansion Method (Multiplicative Expansion)	Number of Expanded Data	Total Number of Expanded Data Sets
Non-estrus	437	2134	a. Brightness enhancement;	1311	6402
Pre-estrus	420		b. Contrast enhancement;	1260	
Mid-estrus	428		c. Random flipping;	1284	
Late-estrus	419		d. Random rotation (with cropping).	1257	
Pseudo-estrus	430		(Expanded 3 times)	1290	

**Table 2:** Core hardware information.

Main Hardware Information	Version
Processor	Intel Core i5-12450H
Graphics Card	NVIDIA GeForce 3060 Laptop
Memory	16 G
Hard Drive	512 G SSD

**Table 3:** Core software information.

Main Software Information	Version
Anaconda	Anaconda3 2019.10 (64-bit)
Python	3.8
CUDA	11.2
Torch	1.8.0
TorchVision	0.9.0
Operating System	Windows11 (23H2)
PyCharm	2021.3
Python	3.8

**Table 4:** Results of ablation experiments.

Algorithms	mAP	F1-Score	Recall	GFLOPs
YOLOv8n (Baseline)	0.908	0.88	0.857	8.9
YOLOv8n + DDTM	0.913	0.89	0.858	8.6
YOLOv8n + DWR	0.919	0.90	0.859	8.3
YOLOv8n + CAFM	0.910	0.88	0.858	9.1
YOLOv8n + DDTM + CAFM	0.916	0.90	0.861	8.5
YOLOv8n + DWR + CAFM	0.922	0.91	0.868	8.4
YOLOv8n + DDTM + DWR + CAFM + CIoU	0.931	0.92	0.873	8.2
YOLOv8n + DDTM + DWR + CAFM + FocalerIoU (ICAE-YOLO)	0.934	0.92	0.879	8.0

**Table 5:** Performance comparison of different models of algorithm.

Algorithms	mAP	F1	Precision	Recall	GFLOPs	Parameters (M)
YOLOv8n	0.908	0.88	0.904	0.857	8.9	5.76
ICAE-YOLO (Ours)	0.934	0.92	0.960	0.879	8.0	4.97
YOLOv5n	0.837	0.79	0.837	0.742	7.2	6.89
YOLOv7tiny	0.896	0.84	0.846	0.832	9.6	8.84
YOLOv9t	0.886	0.87	0.91	0.827	8.5	4.66
YOLOv10n	0.899	0.86	0.834	0.841	8.4	5.18
YOLOv11n	0.912	0.88	0.924	0.844	6.6	5.05
Faster R-CNN	0.832	0.82	0.821	0.730	26.5	31.60

**Table 6:** Performance improvement rate of ICAE-YOLO alongside those of other comparison algorithms.

Algorithms	mAP Enhancement Rate	F1 Enhancement Rate	*p*-Value Improvement Rate	R-Value Improvement Rate	Reduction Rate of GFLOPs	Reduction Rate of Parameters
YOLOv8n	2.863%	4.545%	6.195%	2.567%	10.112%	13.715%
YOLOv5n	11.589%	16.456%	14.695%	18.464%	−11.111%	27.866%
YOLOv7tiny	4.241%	9.524%	13.475%	5.649%	16.667%	43.778%
YOLOv9t	5.418%	5.747%	5.495%	6.288%	5.882%	−6.652%
YOLOv10n	3.893%	6.977%	15.108%	4.518%	4.762%	4.054%
YOLOv11n	2.412%	4.545%	3.896%	4.147%	−21.212%	1.584%
Faster R-CNN	12.260%	12.195%	16.931%	20.411%	69.811%	84.272%

**Table 7:** Average precision of each algorithm alongside those of all classes and mean average precision.

Algorithms	AP_Allclass_	AP_Pseudoestrus_	AP_Notoestrus_	AP_Preoestrus_	AP_Midoestrus_	AP_Lateoestrus_
YOLOv8n	0.908	0.909	0.821	0.976	0.964	0.870
ICAE-YOLO (Ours)	0.934	0.973	0.855	0.984	0.993	0.866
YOLOv5n	0.847	0.848	0.787	0.947	0.831	0.822
YOLOv7tiny	0.896	0.896	0.832	0.948	0.933	0.871
YOLOv9t	0.886	0.887	0.819	0.974	0.906	0.844
YOLOv10n	0.899	0.909	0.850	0.940	0.891	0.905
YOLOv11n	0.912	0.913	0.816	0.979	0.919	0.933
Faster R-CNN	0.832	0.832	0.821	0.813	0.772	0.922

**Table 8:** Details of data collection for model evaluation.

Collection Location	longitude and Latitude	Acquisition Time	Pig Breed	Acquisition Time Span and Associated Light Conditions	Number of Images
Duan Village, Sinnian Township, Fenxi County, Shanxi Province, China	Longitude 111.572259, latitude 36.613203	12 April 2024	I. Yorkshire Hogs (Large White Hogs) × 4 (including 1 pseudo-estrus case) II. Long White Pig × 4 (including 1 pseudo-estrus case)	7:30–11:00 and 15:30–18:00 Daylight and well lit 11:00–15:30 Daylight and too much light 18:00–23:00 Lights and not enough light	309
Pu county in Shanxi province, China	Longitude 111.095996, latitude 36.411902	2 May 2024	Jinfen White Pig × 5 (including 1 pseudo-estrus case)	7:00–17:30 Shade and normal light 17:30–23:00 Lights and not enough light	277

**Table 9:** Comparison of model scores and expert assessment results.

Model	mAP	Recall	F1-Score
Model Prediction	0.921	0.913	0.908
Expert Assessment	0.959	0.940	0.939
Inaccuracies	−0.038	−0.027	−0.031
Error Rates	3.9624%	2.8723%	3.3013%

**Table 10:** Deep learning-based methods developed for recognizing estrus in livestock in recent years.

Research Objectives	Model and Identification Basis	Recognition Effect	Performance		
			mAP	Parameters (M)	Accuracy
Pig Haibo Zheng et al. [23]	FD-YOLOV5s Judged by temperature changes in the vulva of sows	Not in estrus and estrus	99.1%	3.86	-
Pig Kaidong Lei et al. [24]	DBN, SAE, SVM Detecting estrus in weaned sows by simulating the boar’s voice, odor and touch	Not in estrus and estrus	-	-	96.12%; 98.25%; 90.00%
Pig Yuan Wang et al. [25]	MobileNetV3 Resnet Determination of estrus according to the voice of sows in heat	Not in estrus and estrus	97.12%	5.94	-
Cow Zheng Wang et al. [41]	E-YOLO Judging by the cow’s crawling behavior	Not in estrus and estrus	93.90%	3.04	-
Cow Jun Wang et al. [42]	LSTM Judging by cow’s voice characteristics	Not in estrus and estrus	-	-	-
Cow Wang Zheng et al. [43]	YOLO v5n Judging by cow’s estrus behavior	Not in estrus and estrus	97.70%	0.72	-
Buffalo Indu Devi et al. [44]	Judged by barking threshold	Not in estrus and estrus	95.00%	-	-

**Table 11:** Experimental results under different light conditions.

Lighting Conditions	Expert Assessment of Vulva Visibility in Sows	Number of Images	mAP	F1-Score
Normal Light	Excellent	93	0.968	0.959
Bright Light	Poor	89	0.844	0.839
Low Light	Average	90	0.904	0.900

## Data Availability

The authors do not have permission to share data.
